# Orbitofrontal Cortex and the Early Processing of Visual Novelty in Healthy Aging

**DOI:** 10.3389/fnagi.2016.00101

**Published:** 2016-05-02

**Authors:** David A. S. Kaufman, Cierra M. Keith, William M. Perlstein

**Affiliations:** ^1^Department of Psychology, Saint Louis UniversitySt. Louis, MO, USA; ^2^Department of Clinical and Health Psychology, University of FloridaGainesville, FL, USA; ^3^Department of Psychiatry, University of FloridaGainesville, FL, USA; ^4^VA RR&D Brain Rehabilitation Research Center of Excellence, Malcom Randall Veterans Administration Medical CenterGainesville, FL, USA

**Keywords:** ERPs, aging, oddball, orbitofrontal cortex, attention, sLORETA

## Abstract

Event-related potential (ERP) studies have previously found that scalp topographies of attention-related ERP components show frontal shifts with age, suggesting an increased need for compensatory frontal activity to assist with top-down facilitation of attention. However, the precise neural time course of top-down attentional control in aging is not clear. In this study, 20 young (mean: 22 years) and 14 older (mean: 64 years) adults completed a three-stimulus visual oddball task while high-density ERPs were acquired. Colorful, novel distracters were presented to engage early visual processing. Relative to young controls, older participants exhibited elevations in occipital early posterior positivity (EPP), approximately 100 ms after viewing colorful distracters. Neural source models for older adults implicated unique patterns of orbitofrontal cortex (OFC; BA 11) activity during early visual novelty processing (100 ms), which was positively correlated with subsequent activations in primary visual cortex (BA 17). Older adult EPP amplitudes and OFC activity were associated with performance on tests of complex attention and executive function. These findings are suggestive of age-related, compensatory neural changes that may driven by a combination of weaker cortical efficiency and increased need for top-down control over attention. Accordingly, enhanced early OFC activity during visual attention may serve as an important indicator of frontal lobe integrity in healthy aging.

## Introduction

The ability to orient to new stimuli and respond with flexibility to a changing environment is a crucial skill (Barceló et al., 2002). One way to simulate environmental change and examine cognitive control involves the introduction of an unexpected novel stimulus, which intends to capture and divert attention (de Fockert et al., 2004; Barcelo et al., 2006). In cognitive neuroscience, the oddball paradigm has been frequently used to assess neural responses to irregularly occurring, novel stimuli (Friedman, 2003). Although it is a relatively simple task involving response to an infrequent target stimulus presented amongst more common standard stimuli, it has been interpreted as a probe for frontal cortex activity (Fabiani and Friedman, 1995). Huettel and McCarthy (2004) theorized that activation of the prefrontal cortex during the oddball paradigm can be associated with inhibition and modification of behavioral response strategies. Given the infrequency of targets, oddball tasks foster a predominantly passive response style that must be overcome when targets are detected. Accordingly, optimization of attentional processing during these tasks requires synchronization between bottom-up and top-down processing systems.

Top-down processing assists in the voluntary discrimination of relevant stimuli from distractors (Gazzaley et al., 2005). Normal aging is associated with increases in distractibility, which may be due to increased difficulty filtering irrelevant sensory information during attentional processing (McDowd and Filion, 1992; Chao and Knight, 1997; Alain and Woods, 1999; Fabiani et al., 2006). This inhibitory deficit hypothesis is commonly cited as an influential factor driving age-related cognitive decline (Hasher and Zacks, 1988; Gazzaley and D’Esposito, 2007). Age-related changes in attentional control are often linked to frontal lobe structures, which undergo structural and functional compromise in healthy aging (Raz et al., 2005). However, older adults also exhibit declines with bottom-up sensory processing (Carp et al., 2011), which can also contribute to age-related declines in visual attention. While many studies have suggested that enhanced activity in frontal lobe structures helps to compensate for age-related declines in sensory processing (Reuter-Lorenz and Cappell, 2008; Li et al., 2013), it remains unclear exactly how top-down mechanisms of control are coordinated.

The neural time course of attentional processing can be examined with high temporal resolution using electrophysiological methods. Visual oddball studies have frequently reported event-related potentials (ERPs) that highlight age-related differences in attention. In particular, target-related P3 components (also referred to as P300 or P3b) demonstrate increased latency in older adults, while overall amplitudes decrease with age (Polich, 2007; Friedman, 2008). In young adults, target-related P3 amplitudes are typically maximal over parietal electrode sites, yet in older adults these amplitudes decrease at both parietal and central locations, while often preserving higher amplitudes over frontal sites. This has often been interpreted as an age-related “frontal shift” with regard to neural resources necessary for processing attentional targets (Fabiani and Friedman, 1995; Friedman et al., 1993). When infrequent distracters are incorporated into oddball paradigms, they evoke a different type of P3 response (often referred to as P3a or novelty P3), which reflects a more rapid detection of novel, task-irrelevant events (Simons and Perlstein, 1997). Distracter-related P3 amplitudes are maximal over frontocentral sites, and increase when the target recognition is more difficult (Katayama and Polich, 1999; Polich and Comerchero, 2003). Although the shift in target-related P3 amplitudes has often presumed a link with frontal lobe structures, imaging studies suggest that a broader network of neural sources give rise to this component, including prefrontal cortex, temporal-parietal junction, lateral parietal cortex, and anterior cingulate (Bedowski et al., 2004).

While many studies have investigated age-related frontal shifts in P3 potentials, effects of aging on early visual sensory (or “exogenous”) ERPs have received less attention. This omission is important, as recent studies have revealed that frontal lobe structures may influence visual processing in very early time windows (80–150 ms). Using a combination of Magnetoencephalography (MEG) and Functional magnetic resonance imaging (fMRI) methods, Bar et al. (2006) found that the orbitofrontal cortex (OFC) exhibited differential activity that predicted successful recognition of visual objects, beginning approximately 80 ms after the object appeared. These authors took their findings to suggest that the OFC may receive coarse visual inputs following initial sensory processing which can then be used to make predictions that guide subsequent visual processing that is more specialized (Bar, 2009). Indeed, some cells in the OFC appear to be particularly sensitive to visual novelty, showing selective activation between 80–120 ms post-stimulus (Rolls et al., 2005). While few studies have reported age-related changes that occur in these early sensory processing stages, De Sanctis et al. (2008) observed increased N1 amplitude in older adults, along with enhanced early frontal cortical activation. These results suggest that older adults may enhance frontal activity during early visual processing in order increase their top-down control over visual attention.

In the current study, a three-stimulus oddball paradigm was used to examine early processing of visual novelty in young and older adults. Scalp ERP results were analyzed for early sensory processing and subjected to source localization. Distracter novelty was manipulated during the task, which presented either monochrome or colorful distractors in different trial blocks. Color distracters were presented in order to evoke early visual cortex activity associated with color feature selection, which can be observed as a large posterior negativity, beginning approximately 140 ms post-stimulus and localized to occipital cortex (Hillyard and Anllo-Vento, 1998). Given the proposed role of the OFC in novelty processing and top-down regulation of attention, it was hypothesized that this structure would become differentially involved during the early processing of distracters (80–120 ms). In particular, this early OFC activation was expected to precede visual cortex activations associated with color feature processing. In line with prior studies reporting age-related decline in visual attention, older adults were expected to rely on greater levels of OFC involvement during early processing of colorful distracters. In addition to the oddball paradigm, participants completed several neuropsychological tasks, so that age-related changes could be interpreted in the broader context of executive functioning.

## Materials and Methods

### Participants

A total of 34 participants (20 younger, 14 older) were recruited from advertisements in the local community and undergraduate psychology courses. Demographic characteristics are presented in Table 1. Participants were matched across all characteristics except for age, in which younger participants had a mean age (± SD) of 22.1 years (± 3.1) and older participants had a mean age of 64.4 years (± 9.5). Neuropsychological testing revealed that older adults had comparable levels of cognition (MMSE), inhibition (Stroop), and feedback-dependent problem solving (Wisconsin Card Sorting Test). However, older adults were significantly slower on tasks of complex attention and set-shifting (Digit Symbol Coding, Trails A and B). Potential participants (3 young, 1 older) were excluded from the study because they endorsed a history of substance abuse or dependence, acquired brain disorders (e.g., traumatic brain injury), neurological disorders, or color-blindness. Participants provided written informed consent according to procedures established by the University of Florida Health Science Center Institutional Review Board and were provided either $20 or course extra credit for study participation.

**Table 1:** **Demographic and cognitive data for younger and older participants**.

	Younger (*n* = 20)		Older (*n* = 14)		
	Mean	(SD)	Mean	(SD)	*p*
**Demographics**
Age (years)	22.1	(3.1)	64.4	(9.5)	*<0.001*
Education (years)	15.4	(1.7)	17.1	(3.3)	ns
Female (%)	50	–	43	–	ns
Right-handed (%)	85	–	86	–	ns
**Cognitive functioning**
MMSE	28.9	(1.1)	28.7	(1.3)	ns
Digit symbol coding (Total)	87.7	(12.2)	77.4	(11.7)	*0.02*
Stroop word reading (sec)	101.4	(15.0)	99.5	(13.5)	ns
Stroop color naming (sec)	80.0	(14.0)	72.6	(15.9)	ns
Stroop color word naming (sec)	46.9	(12.6)	38.8	(11.9)	ns
Trails A (sec)	20.8	(4.7)	27.7	(10.6)	*0.01*
Trails B (sec)	47.0	(15.4)	63.8	(28.7)	*0.03*
WCST Categories completed	6.0	0.0	5.6	(1.3)	ns
WCST Total errors	15.1	(9.6)	17.6	(16.7)	ns
WCST Perseverative responses	8.6	(4.3)	10.3	(10.4)	ns
WCST Perseverative errors	7.9	(3.8)	9.8	(8.9)	ns
WCST Set failure	0.1	(0.3)	0.3	(0.7)	ns

*MMSE, Mini-Mental State Examination; WCST, Wisconsin Card Sorting Test*.

### Event-Related Potential Stimuli and Task

**Figure 1:**
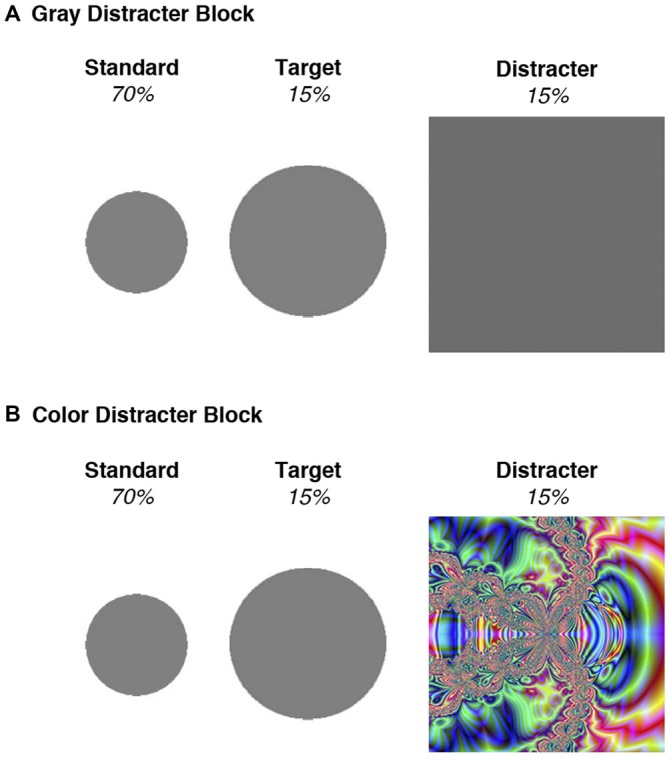
**Stimuli used in the two blocks of the oddball task. (A)** All stimuli in the gray distracter block were comprised of an identical gray color. **(B)** Standards and targets in the color distracter block were identical to those presented in the gray distracter block, but the distracters consisted of unique colorful fractal designs.

Stimuli and procedures for the three-stimulus oddball task were adapted from Polich and Comerchero (2003). Standard stimuli, to which the participants were told not to respond, consisted of small gray circles measuring 2” in diameter. Target stimuli were medium-sized gray circles, measuring 3”, and participants were instructed to press a button when they saw these stimuli. Distracter stimuli, to which the participants were told not to respond, consisted of large squares measuring 4.5” in diameter. Because distracters were deviant from standards and targets in these two characteristics (size, shape), the amount of visual attention devoted to these distracters was optimized (Polich and Comerchero, 2003; Sawaki and Katayama, 2007). Distracter novelty was further manipulated by stimulus features contained within the large squares. Half of the distracters were gray, like the targets and standards, and were identical in appearance throughout the experiment. The other distracters contained colorful fractal designs that were unique and only occurred once over the course of the experiment (see Figure 1 for an example). Mean (± SD) luminance intensity for the color distracter slides were 24.1 cd/m^2^ (± 9.3), which did not deviate substantially from that of gray distracters (23.1 cd/m^2^). Thus color and gray distracters had similar physical properties (size, luminance), despite their differences in chromatic features.

A total of 600 stimuli were randomly presented in four blocks of 150. Seventy percentage were standard stimuli (small circles), 15% were targets (medium circles), and 15% were distracters (large squares). Each stimulus was presented for 75 ms, with a 2 s inter-stimulus interval. Color and gray distracters were presented in different trial blocks to examine the block-related effects of distracter type on behavioral and electrophysiological data. Presentation order of color and gray distracter blocks was counterbalanced across participants such that half of the participants completed the color distracter blocks first and third, while the other participants completed color distracter blocks second and fourth. A practice task consisting of 10 stimuli (6 standards, 2 targets, and 2 gray distracters) was presented in advance to ascertain that all participants were able to discriminate targets from standards. The practice task was repeated as needed until all participants achieved 8 out of 10 correct responses.

### Electrophysiological Data Recording, Reduction, and Measurement

Electroencephalographram data were recorded from the scalp using a 64-channel system (Electrical Geodesics, Inc., Eugene, Oregon) running Net Station Software. Impedance of electrodes was maintained below 50 kΩ. EEG was initially referenced to the vertex and recorded continuously at 250 Hz, with on-line band-pass filtering from 0.1 to 100 Hz. Electroencephalogram data were then re-referenced to an average reference off-line and digitally low-pass filtered at 30 Hz. Eye movement and blink artifacts were corrected using spatial filtering methods (Scherg, 1990). The mean (± SD) amplitude used for rejection was a lenient 110 μV (± 16), in order to maximize the number of trials that could be included in averaging without introducing artifacts. The maximum allowable amplitude settings for each trial were individualized for each participant (as recommended by Luck, 2005), but did not differ by group, *t*_(32)_ = 0.25, *p* > 0.80. Point-to-point transitions were not allowed to exceed 75 μV. Of the 64 electrodes used to collect data, less than 2% were interpolated during analysis in order to correct for artifacts.

Individual-subject stimulus-locked averages were derived separately for standard, target, and distracter stimuli in each of the two distracter blocks. Error trials were excluded from ERP analysis. Epochs were extracted from a window of 200 ms prior- to 800 ms post-stimulus presentation and baseline-corrected (200 ms prior to stimulus onset) before subject averaging and analysis. Four occipital electrodes corresponding with O1, O2, PO7, and PO8 were used to examine early posterior positivity (EPP; EGI sensors: 32, 37, 40, 45).

Early Posterior Negativity (EPN) was maximal over medial occipital sites, particularly for younger adults, so three medial occipital electrodes corresponding with O1, O2, and Oz were used to quantify this component (EGI sensors: 37, 40, 38). Four midline electrodes corresponding with FCz, Cz, CPz, Pz were chosen for measurement and analysis of P3 responses (EGI sensors: 4, 65, 30, 34). ERP components were analyzed using adaptive mean amplitude and peak latency scoring protocols in Net Station Software. The adaptive mean algorithm identified a peak within each selected time window and then defined a new time window around this peak for scoring the mean voltage. Time windows used for scoring the different ERP components were as follows: Lateral EPP: 80–130 ms (adaptive mean: 8 ms pre- to 8 ms post-peak), Midline EPN: 100–220 ms (adaptive mean: 8 ms pre- to 8 ms post-peak), and P3: 300–650 ms (adaptive mean: 120 ms pre- to 120 ms post-peak).

### Data Analysis

Visual inspection of the oddball task data for normality revealed a considerable positive skew. We, therefore, applied the arcsine correction for all error rate data (Neter et al., 1985) and subjected them to a 2-Distracter (gray, color) × 3-Stimulus (standard, target, distracter) repeated measures analysis of variance (ANOVA). For analyses of oddball task response time (RT), median RTs were employed for correct responses (Ratcliff, 1993) and compared between distracter conditions via dependent sample *t*-tests.

Because of their broad scalp distribution, P3 mean amplitudes and peak latencies were examined with 2-Distracter (gray, color) × 3-Stimulus (standard, target, distracter) × 4-Electrode Site (Fcz, Cz, Cpz, Pz) repeated measures ANOVAs. EPP and EPN components were subjected to 2-Distracter (gray, color) × 3-Stimulus (standard, target, distracter) repeated measures ANOVAs, using data from the average electrodes in which each ERP component showed maximal amplitude (Picton et al., 2000). Orthogonal (“Helmert”) planned contrasts were used in these ANOVAs such that stimulus comparisons were made in the following order: (1) standards vs. targets and distracters; and (2) targets vs. distracters. When applicable, difference waves were calculated by subtracting one condition of interest from another (e.g., color distracters—gray distracters). In order to isolate scalp ERP signals associated with color distracter processing, difference waves were calculated for each stimulus type, such that trials from the gray distracter block were subtracted from trials in the color distracter block.

When necessary to decompose ANOVA results, follow-up *post hoc* comparisons were made using Bonferroni corrections for multiple comparisons. The Huynh-Feldt epsilon adjustment was used for all repeated measures ANOVAs with greater than 1 degree of freedom; uncorrected degrees of freedom and corrected *p*-values are reported.

### Neural Source Analysis

Source localization was examined using a realistic finite difference forward model of head conductivity (based on a typical adult head MRI from the Montreal Neurological Institute). A dense dipole set was used in GeoSource 2.0 Software (Electrical Geodesics Inc.), which includes 2447 source dipoles (*x*, *y*, *z* triples) in 7 mm^3^ voxels of gray matter, following the method of Pascual-Marqui et al. (1994). The linear inverse solution was computed using the constraints of sLORETA.

Brodmann areas (BAs) were examined using “source montages” defined by the Talairach coordinates (Waters and Tucker, 2013). All dipoles in the brain were represented by a triple regional source, consisting of three dipoles in the *x*, *y*, and *z* orthogonal orientations. The net equivalent dipole for each BA source region was computed as the vector sum of the *x*, *y*, *z* triples for the voxels contained within that region. Particular BAs were selected for statistical analysis based on our predictions that OFC and visual cortex would both be active during the early time windows corresponding to scalp ERP components. Thus BA 11 and BA 17 were selected as* a priori* regions of interest, with neural time course activations for these two regions extracted as the root mean square (RMS) of the *x*, *y*, *z* orthogonal orientations. Consistent with other methods used to examine neural source effects (Lamm et al., 2013; Waters and Tucker, 2013), these two source regions were noted to exhibit changes in source intensity (above an arbitrary threshold) that was observably coincident with the temporal features of the scalp ERP. Mean amplitudes from these source waveforms were then calculated for particular time windows of interest (100 ms, 140 ms), and subjected to difference wave calculation in a manner similar to scalp ERP components of interest.

## Results

### Oddball Task Performance

Accuracy and RT data from the oddball task were subjected to separate 2-Distracter × 3-Stimulus ANOVAs. With regard to task accuracy, planned contrasts revealed a main effect of distracter type, such that participants made more errors to infrequent stimuli than standards, [*F*_(1,32)_ = 47.8, *p* < 0.001, *η*^2^ = 0.60], while distracters evoked more errors than targets [*F*_(1,32)_ = 18.3, *p* < 0.001, *η*^2^ = 0.36]. There was no main effect of group for accuracy. Errors were much more common for trials in the color distracter blocks than gray distracter blocks [*F*_(1,32)_ = 69.4, *p* < 0.001, *η*^2^ = 0.69], with high error rates for distracters (21.0%), followed by targets (6.1%), and standards (2.0%) in the color distracter block. With regard to target RT, there were no main effects of group or distracter type or Group × Distracter interaction.

### Event-Related Potential Data

**Figure 2:**
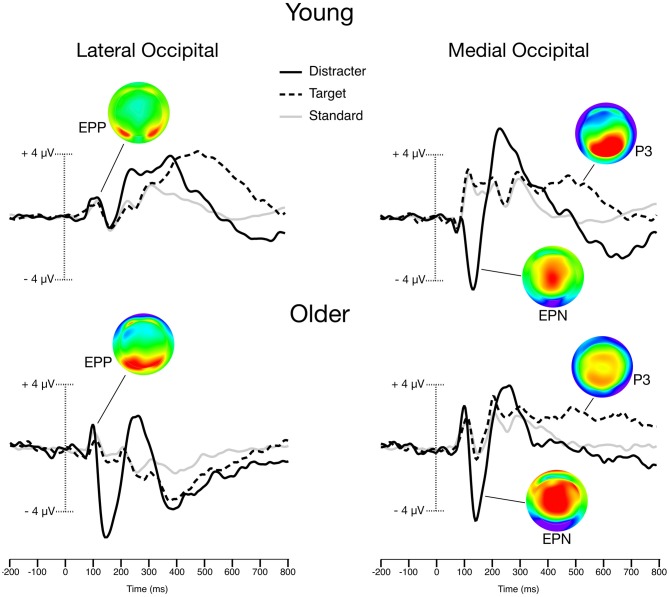
**Grand-averaged Event-related potentials (ERPs) from the color distracter block trials.** Scalp maps illustrate the peak amplitudes of color distracter processing for early posterior positivity (EPP; 100 ms), Early Posterior Negativity (EPN; 140 ms), and P3 (400 ms). Lateral occipital waveforms were averaged from O1, O2, PO7, and PO8 electrodes. Medial occipital waveforms were averaged from O1, O2, and Oz electrodes.

Within the gray distracter block, standard stimuli waveforms contained an average (± SD) of 165.5 ± 22.9 trials, while target waveforms contained 35.5 ± 5.1 trials and distracter waveforms contained 34.0 ± 4.9. Within the color distracter block, standard stimuli waveforms contained an average (± SD) of 165.7 ± 23.9 trials, while target waveforms contained 34.8 ± 5.2 trials and distracter waveforms contained 34.6 ± 5.2. A Group (2) × Distracter (2) × Stimulus (3) ANOVA confirmed that younger and older participants did not differ in their number of trials that were acceptable for analysis. Furthermore, distracter type did not influence the number of trials comprising the waveforms (main effect and interaction with stimulus type were nonsignificant). Stimulus-locked ERP waveforms taken from lateral and medial occipital sites can be seen in Figure 2.

#### Early Posterior Positivity (EPP)

EPP activity was maximal over lateral occipital sites. While there was no main effect of group, planned contrasts revealed that EPP amplitude exhibited a significant Group × Distracter × Stimulus interaction. Older adults exhibited larger EPP for color distracters than younger adults, even though other stimuli showed no group differences, *F*_(1,32)_ = 8.9, *p* < 0.01, *η*^2^ = 0.22.

**Figure 3:**
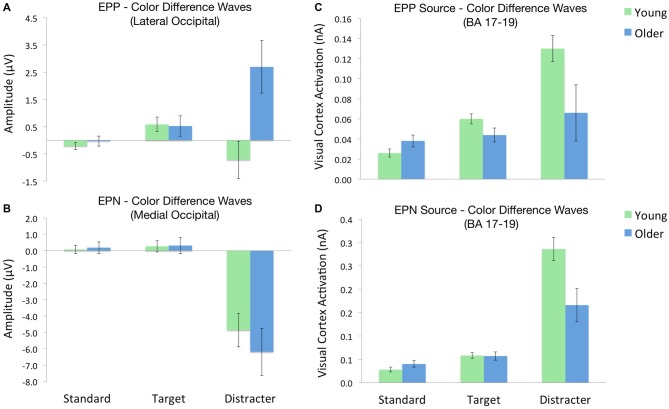
**ERP amplitudes and source activations across each oddball stimulus.** Each value depicts the contrast between stimuli in the color and gray distracter blocks (i.e., color-gray difference waves). **(A)** EPP amplitudes across lateral occipital electrodes. **(B)** EPN amplitudes across medial occipital electrodes. **(C)** Visual cortex source activity during the EPP time window. **(D)** Visual cortex source activity during the EPN time window.

In order to better isolate scalp ERP signals associated with color distracter processing, difference waves were calculated for each stimulus type, such that trials from gray distracter blocks were subtracted from those with color distracters. This resulted in the following EPP difference wave categories: (1) standard color-gray; (2) target color-gray; and (3) distracter color-gray. A Group (2) × Stimulus (3) ANOVA confirmed that the color-related enhancement of distracter EPP was only seen in older adults, *F*_(1,32)_ = 15.7, *p* < 0.001, *η*^2^ = 0.33. Figure 3A shows these effects by graphing the standardized EPP amplitudes (i.e., color-gray difference waves) for each stimulus across groups.

EPP latencies peaked between 100–112 ms across conditions. There was a significant main effect of group, such that EPP amplitudes peaked earlier for older adults, *F*_(1,32)_ = 4.3, *p* < 0.05, *η*^2^ = 0.12. A Distracter × Stimulus interaction revealed that EPP peak latency was shorter for color distracters than gray, while other stimuli did not differ as a function of distracter block, *F*_(1,32)_ = 6.1, *p* < 0.05, *η*^2^ = 0.16.

#### Early Posterior Negativity (EPN)

EPN activity was maximal over medial occipital sites. There was a main effect of group, with older adults exhibiting larger EPN than younger adults, *F*_(1,32)_ = 12.8, *p* = 0.001, *η*^2^ = 0.29. Planned contrasts revealed that EPN amplitude exhibited significant a distracter × stimulus type interaction, such that color distracters evoked larger EPN than gray distracters, *F*_(1,32)_ = 25.1, *p* = 0.001, *η*^2^ = 0.44. However, this interaction did not differ as a function of group, and no other effects on EPN were significant.

EPN difference waves were calculated for each stimulus type, following the same procedures employed for EPP difference waves. A Group (2) × Stimulus (3) ANOVA confirmed a strong main effect of stimulus, such that color distracters evoked larger EPN amplitudes than targets or standards, *F*_(1,32)_ = 43.3, *p* < 0.001. This color-related enhancement of distracter EPN did not differ as a function of group, *F*_(1,32)_ = 0.1, *p* > 0.72. Figure 3B shows these effects by plotting the standardized EPN amplitudes (i.e., color-gray difference waves) for each stimulus across groups.

EPN latencies peaked between 140–160 ms across conditions. Planned contrasts revealed a Distracter × Stimulus interaction, such that EPN peak latency was shorter for distracters than targets and standards, but only in color distracter block, *F*_(1,32)_ = 6.7, *p* < 0.05, *η*^2^ = 0.17. Thus EPN latency did not vary as a function of group.

#### P3

P3 activity had a broad distribution and could be seen across multiple midline sites. Planned contrasts revealed a significant Group (2) × Distracter (2) × Stimulus (3) × Site (4) interaction, such that older patients exhibited larger centrofrontal P3 amplitude for targets and distracters relative standards in the color distracter block [*F*_(1,32)_ = 10.0, *p* < 0.01, *η*^2^ = 0.24]. This effect can be visualized when comparing the P3 voltage distribution maps in Figure 2, which demonstrate enhanced centrofrontal P3 amplitude for older adults relative to younger controls.

Midline P3 latencies peaked between 380–450 ms across conditions. Planned contrasts of peak latencies revealed a main effect of stimulus, such that target P3 latencies were longer than those evoked by distracters [*F*_(1,32)_ = 14.4, *p* = 0.001, *η*^2^ = 0.29], or standards [*F*_(1,32)_ = 6.7, *p* < 0.05, *η*^2^ = 0.17]. While there was no main effect of group, group interacted with stimulus [*F*_(1,32)_ = 13.1, *p* = 0.001, *η*^2^ = 0.29], such that older adults had P3 latencies that were longer for infrequent stimuli (targets and distracters) relative to young adults.

#### Neural Source Modeling

**Figure 4:**
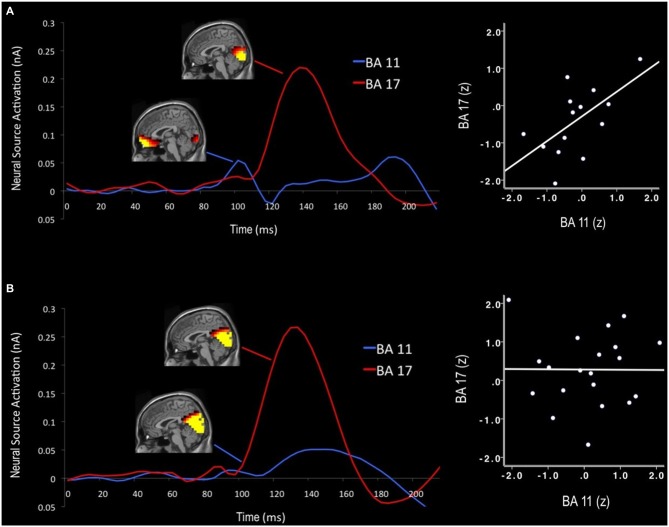
**Neural time course of distracter color-gray difference waves for older (A) and younger adults (B).** Activation maps display cortical activity at 100 ms and 140 ms that was preferential for color-related distracter processing. Early orbitofrontal cortex (OFC; BA 11) activations predicted subsequent visual cortex activity (BA 17) in older adults, but not for younger adults. Note: Scales visible source activations differ for each time window (100 ms: visible range = 0.06–0.10 nA; 140 ms: visible range = 0.15–0.25 nA).

In order to estimate neural sources associated with the early sensory effects of color distracters, difference waves for each stimulus were subjected to sLORETA analysis. Visual examination of sLORETA source models revealed distracter-related activations in visual cortex (BA 17) and OFC (BA 11) regions within the first 200 ms post-stimulus. For each BA region of interest, the RMS of each dipole’s three orientations was calculated, as shown in the source waveforms in Figure 4. For both age groups, activity began to increase in visual cortex regions approximately 80 ms post-stimulus, peaked around 140 ms, and then decreased back down to baseline around 200 ms post-stimulus. In order to quantify these activations, mean source amplitudes were scored for visual cortex ROIs during the beginning (100 ms) and peak activation (140 ms) portions of this time window. It should be noted that this early time window corresponds to the peak latency of the scalp EPP, while the later time window corresponds to the peak latency of the scalp EPN. Shortly after the initial increase in visual cortex activity, small activations can be seen in OFC between 90–110 ms post-stimulus. Accordingly, mean source amplitudes were scored for the OFC during the peak of this frontal activation (100 ms), which also overlaps in time with the scalp EPP. No distinct neural activations were evoked by color distracters during the P3 time window (350–600 ms); thus, no source models were generated for P3 components.

Cortical source activations for each stimulus were then subjected to difference wave contrasts (color-gray distracter), in a manner similar to EPP and EPN scalp waveforms. Activations from BA 17 were subjected to separate Group (2) × Stimulus (3) ANOVAs for the early (100 ms) and later (140 ms) time windows of visual processing. Visual source activations in the EPP time window (100 ms) revealed a Group × Stimulus interaction, such that older adults exhibited decreased visual cortex activity for color distracters relative to younger adults, despite the fact that standards and targets evoked equal activity between groups, *F*_(1,32)_ = 8.1, *p* < 0.01, *η*^2^ = 0.20. Likewise, the EPN time window (140 ms) also showed this interaction, with younger adults exhibiting greater visual cortex activity for color distracters, *F*_(1,32)_ = 5.5, *p* < 0.05, *η*^2^ = 0.15. Thus despite the fact that color distracters evoked larger scalp EPP amplitudes in older adults, visual cortex activations were stronger in younger adults during both EPP and EPN time windows, as shown in Figures 3C,D.

Neural source activations were found to be associated with scalp ERP components, but the relationships between EPP and source activity differed as a function of age. Older adults showed a positive correlation between early primary visual cortex activity (100 ms) and color distracter EPP (*r* = 0.60, *p* < 0.05), such that stronger visual cortex activity correlated with larger scalp signals for color distracters. Additionally, early OFC activation (100 ms) correlated with EPP amplitude in older adults (*r* = 0.69, *p* < 0.01). In this same time window, younger adults showed no relationships between EPP and neural sources in the visual cortex or OFC. EPN amplitude was correlated with visual cortex activations (140 ms) similarly for older (*r* = −0.64, *p* < 0.05) and younger adults (*r* = −0.68, *p* = 0.001).

Neural source activations from OFC were also subjected to Group (2) × Stimulus (3) ANOVA to examine the effects of color distracters. Unlike visual cortex regions, the strength of source signal in the OFC did not vary significantly as a function of group, *F*_(1,32)_ = 0.54, *p* > 0.46, and there were no Group × Stimulus interactions, *F*_(1,32)_ = 0.16, *p* > 0.68. However, older adults showed a significant relationship between early OFC and visual cortex activity, such that early (100 ms) OFC activations correlated with subsequent (140 ms) visual cortex activations (*r* = 0.61, *p* < 0.05). Young adults did not show this association. RMS source waveforms extracted from dipoles in these regions reveal the neural time course of OFC and visual cortex activity, as seen in Figure 4. Older adults exhibited an early peak of OFC activation approximately 100 ms post-stimulus, followed by a subsequent peak of visual cortex activation approximately 140 ms post-stimulus. Younger adults did not show this early OFC activation or any association between OFC and visual cortex during early visual processing.

#### Correlations with Executive Function

In older adults, several scores from several neuropsychological tests were associated with scalp ERPs and source activations. For older adults, the total number of errors on the WCST was positively correlated with color distracter EPP amplitude (*r* = 0.60, *p* < 0.05). Early OFC activations (100 ms) correlated with scores from Digit Symbol Coding (*r* = 0.67, *p* < 0.01) and Stroop Color Word Naming (*r* = 0.54, *p* < 0.05). Additionally, subsequent visual cortex activations (140 ms) correlated with Digit Symbol Coding (*r* = 0.76, *p* < 0.01). Thus better executive function in older adults was associated with enhanced early processing of color distracters—larger scalp EPP, enhanced OFC activations (100 ms), and enhanced visual cortex activations (140 ms).

Younger adults showed a different pattern of relationships between neuropsychological measures and color distracter processing. No tests were correlated with scalp ERPs in young adults. Early visual cortex activations (100 ms) correlated with scores from Stroop Color Word Naming (*r* = 0.54, *p* < 0.05), while subsequent visual cortex activations (140 ms) correlated with completion time from Trails B (*r* = −0.45, *p* < 0.05). OFC activations were not associated with any of the neuropsychological measures. Therefore, better executive function in younger adults was correlated with enhanced processing of color distracters in the visual cortex, but not the OFC.

## Discussion

This study examined age-related differences in visual novelty processing. Occipital electrodes revealed two early sensory ERPs that were selectively enhanced by colorful distracters in the oddball task. An EPP peaked at 100 ms in lateral occipital electrodes, while an early posterior negativity (EPN) peaked at 140 ms in medial occipital electrodes. Enhancement of the EPN was expected, since this component is associated with color feature processing (Hillyard and Anllo-Vento, 1998). However, older adults also exhibited amplified sensory processing of colorful distracters prior to the EPN. This occurred during an earlier stage of visual attention (100 ms after stimulus onset), and was associated with increased activity in primary visual cortex and OFC. Older adults’ OFC activity during this early time window was correlated with subsequent visual cortex activity (140 ms), suggesting that the OFC may have been engaged to help facilitate visual recognition of the colorful distracters. In contrast, young adults did not exhibit any early (100 ms) enhanced processing in scalp EPP or neural source activity. Cells in the OFC have been shown to respond selectively to novel visual stimuli, as early as 80 ms following stimulus onset (Rolls et al., 2005). The function of this early activity may be to direct attention toward unexpected or salient stimuli that require further processing (Petrides et al., 2002). When incoming sensory information is ambiguous, perceptual decisions may benefit from “predictive coding”, in which the brain maintains an internal template in order to better anticipate forthcoming perceptions (Summerfield et al., 2006). Consistent with this view, the OFC appears to be synchronized with early (80 ms) and later (130 ms) visual cortex processing (Bar et al., 2006), in order to enhance processing in sensory cortex and improve recognition success. Until recently, the neuroanatomical connections necessary to explain this effect have not been clear. However, it appears that magnocellular pathways provide rapid feed-forward (occipital to OFC) and feed-backward (OFC to fusiform) pathways on a timescale that would facilitate object recognition (Kveraga et al., 2007). It has also been proposed that a subcortical route may project visual information to the OFC from the pulvinar complex, which receives input directly from the retina (Pessoa and Adolphs, 2010). With either pathway, it would appear that early projections reaching the OFC within 100 ms would provide only coarse visual information (e.g., limited spatial frequency and contrast). However, even this basic level of representation appears to be sufficient to activate a minimal set of perceptual expectations in the OFC, which can then influence subsequent bottom-up visual processing to facilitate recognition (Bar et al., 2006; Kveraga et al., 2007).

In the later stage of color feature processing (140 ms), EPN amplitude was amplified for both groups in response to colorful distracters, which appears to have been driven by broad increases throughout primary and secondary visual cortices (BA 17–19). Young adults had greater activation in visual cortex during this stage of processing than older adults. Weaker ERP correlates of visual processing have been observed in other visual detection studies (Czigler et al., 2006), and support a general notion that visual processing isless efficient in healthy aging. Thus the current findings support prior claims that healthy aging is associated with neural dedifferentiation of visual processing networks (Grady et al., 1994; Madden et al., 2004; Carp et al., 2011; Burianova et al., 2013).

In line with prior oddball studies focusing on P3 potentials, older adults demonstrated a “frontal shift” in their P3 responses to targets and distracters. In older adults, colorful distracters evoked larger P3 amplitudes in centrofrontal sites, even though their anterior P3 responses had longer latencies than young adults. This finding has been documented using ERP methods (e.g., Fabiani and Friedman, 1995), as well as functional neuroimaging (Davis et al., 2008). One implication of these findings is that the anterior shift in neural recruitment reflects compensation to offset age-related declines in posterior brain function. Frontal regions like the OFC may become active earlier in older adults in order to enhance top-down organization of sensory information, which becomes less efficient with age (Burianova et al., 2013).

The involvement of OFC in early processing of visual novelty has interesting implications for our understanding of cognitive aging. While generally appreciated for its role in emotional processing and decision-making (Bechara et al., 2000), the OFC plays a broader role in generating expectations about incoming information (Petrides et al., 2002). Additionally, such findings may be relevant to the inhibitory deficit hypothesis of cognitive aging. While often associated with emotional control over behavior, the OFC plays a prominent role in regulating inhibitory control (Bari and Robbins, 2013). Recent studies have supported a role for OFC in executive functions needed to dissociate between two perceptually similar actions during response conflict (Bryden and Roesch, 2015). In particular, dopamine regulation of OFC may play a critical role in facilitating proper inhibitory control when behavioral flexibility is required (Cheng and Li, 2013).

It is noteworthy that the older adults in the current study were not cognitively impaired. They did not differ from young adults in overall cognitive status or general levels of executive functioning, yet their early OFC activity appeared to influence subsequent bottom-up visual processing. Given the correlations between neural source activity and cognitive tests, it appears that greater OFC involvement in top-down regulation of attention was associated with broader neuropsychological benefits in healthy aging. Unlike the older group, young adults did not show relationships between early OFC activity and executive function, even though early visual cortex activations were associated with better executive function. Due to the greater activity of visual cortex in this group, it is likely that their visual recognition processing was more efficient and needed less compensatory assistance from the OFC. Older adults relied more on the OFC to provide predictive coding at the earliest stage of visual processing, such that they could perform the task successfully despite weaker visual cortex activations. Importantly, this tendency in our older adult sample appeared to be adaptive, as greater levels of this OFC activation was correlated with greater levels of executive function.

In summary, the current study revealed age-related differences in the early sensory processing of novel stimuli. Older adults had enhanced scalp signals corresponding to an early stage of visual processing (100 ms), which correlated with neural activity in both primary visual cortex and OFC. Consistent with prior findings on posterior cortex dedifferentiation, older adults required enhanced OFC activation in order to compensate for reduced visual processing efficiency. This early compensatory OFC activity in older adults predicted subsequent feature processing in visual cortex (140 ms) and was associated with greater levels of complex attention and executive function. Thus older adults benefited from greater top-down control during visual processing. These findings lend support for models of cognitive aging that account for both neural dedifferentiation and compensatory neural reorganization. Future studies are needed to explore the underlying neural dynamics and connectivity between the OFC and visual cortex, and determine the degree to which these findings generalize to older individuals with more significant levels of neural dedifferentiation or cognitive decline.

## Ethics Statement

The study was approved by the University of Florida, Health Sciences Institutional Review Board.

## Author Contributions

DASK, Data collection, analysis, manuscript writing; CMK, Manuscript writing; WMP, Manuscript writing and oversight.

## Funding

This work was supported by Grants from the NIH (T32-AG020499 and R21-NS079767).

## Conflict of Interest Statement

The authors declare that the research was conducted in the absence of any commercial or financial relationships that could be construed as a potential conflict of interest.
